# Nocturnal playback experiments: The response of two European species of birds to singing of foreign male at night

**DOI:** 10.1371/journal.pone.0313427

**Published:** 2024-11-25

**Authors:** Kinga Buda, Jakub Buda, Michał Budka

**Affiliations:** 1 Department of Behavioural Ecology, Adam Mickiewicz University in Poznań, Poznań, Poland; 2 Department of Animal Taxonomy and Ecology, Adam Mickiewicz University in Poznań, Poznań, Poland; Rutgers The State University of New Jersey, UNITED STATES OF AMERICA

## Abstract

Recent studies have shown that nocturnal singing in diurnal birds is a common phenomenon, however, the understanding of the mechanisms, functions and consequences of this behaviour has been lacking. We focused on the night singing of two diurnal songbirds–the yellowhammer and the common chaffinch that are widely distributed in Europe. We conducted day and night playback experiments, during which we broadcast songs of an unfamiliar male to the territory holder at two different stages of the breeding season, to examine whether the night singing in species which normally do not sing at night elicits responses from their conspecifics. We hypothesized that if nocturnal singing had no function, birds would ignore the night-time playback and respond only to the daytime intrusion. Otherwise, a response to the night-time playback would suggest that night singing may increase reproductive success but is limited by ecological factors. We found that, in contrast to the diurnal experiment, neither species responded vocally to the nocturnal playback. In yellowhammers, the probability of flights was higher during and after the playback than before it. This pattern was similar both during the day and night and did not differ between the stages of the season. For the common chaffinches, the probability of flight was low at night and constant across treatments, in contrast to the day when we observed more flights during the playback stage than before and after it. The playback of either species’ songs at night caused the approach of predators, which was not observed during the day. The observed discrepancy in the probability of flights between the species suggests that nocturnal singing is a functional trait that affects other individuals in a specific context. Nocturnal singing does not seem to be a simple behaviour that extends during the day; it is a complex mechanism potentially dependent on specific conditions related to intraspecific communication, predatory pressure, local female access, or species’ evolutionary history. We emphasise that those ecological and evolutionary factors need to be taken into account to understand this phenomenon widely.

## Introduction

Acoustic communication is an effective way of transferring information from sender to receiver over long distances, independent of line of sight or light conditions [1, 2]. Many species have an elaborate system of acoustic communication involving songs–complex vocalisations mainly produced by males and, less often, by females. The two main functions of songs are territory defence and mate attraction [3]. The amount of singing depends on social context (i.e. presence or absence of the female in the territory) and hormones (specifically testosterone [4]). The intensity of vocalising also relies on the stage of the breeding season [5] and the circadian clock [6]. In temperate regions, birds singing is usually limited to 3–4 months of the breeding season. However, even within this short period, seasonal peaks of males’ vocal activity are observed, and in some species, it reflects the current reproductive stage of their mates [7]. For example, males of the house wren (*Troglodytes aedon*) begin singing earlier in the day when they are building nests and when females are fertile than during later nesting stages [8]. In the collared flycatcher (*Ficedula albicollis*), the singing rate is not associated with the period of female fertility [9]. Great tit males sing at high levels during the egg-laying and incubation periods, while blue tit males strongly reduced singing activity after the first days of egg-laying by their females [10]. Another study on the great tit shows that singing occurs most often when the female is not present in the territory [11]. It is a strong case for one of the functions of song being the purpose of maintaining or establishing the pair bond between the male and female.

Most bird species vocalise during the day, with two distinguishable peaks of the singing activity: around sunrise (dawn chorus) and sunset (dusk chorus). These two periods are characterised by specific weather conditions, such as high humidity and lack of strong wind, which support effective sound transmission [12]. Additionally, low air temperature in the morning reduces insect activity, promoting birds to focus on vocalising rather than foraging [13]. However, several studies have shown that some bird species sing at atypical times of the day. For instance, high vocal activity during the day has been observed intermittently in a typically nocturnal species–the common corncrake (*Crex crex*) [14]. It is known that typically diurnal bird species also sing at night. La (2012) showed that night vocalisation by diurnally active birds occurs in at least 51 of the 82 analysed families and 18 of the 22 orders in North America. A similar pattern has been found in central Europe, where nocturnal singing by diurnal birds occurred in 15 of the 32 families and 7 of the 12 analysed orders; however, this behaviour was observed more often in open areas than in forests [15]. Both studies suggest that species can be grouped with respect to singing at night as: (1) occasional singers, (2) regular but not intensive singers, and (3) regular and very intensive singers.

While nocturnal singing by diurnal birds in temperate regions occurs quite often, in Afrotropical highlands this behaviour seems to be very rare. Of the 50 typically diurnal species, only three sang during astronomical night, producing a total of 10 songs during the breeding season [16]. It is hypothesised that tropical birds undertake less risky behaviour than temperate birds [17], which may be reflected in differences in life history between species inhabiting these regions. This raises two important questions: why nocturnal singing by diurnal birds evolved in some groups of birds, geographic regions, or ecological and environmental conditions, while in others it did not, and what the function, if any, of such behaviour is.

Mate attraction and maintaining the pair bond and rival deterrence are the main functions of birdsong. However, the dominant function might vary depending on the species and could change across different times of the day, season, or contexts [18]. In the case of the night singing by diurnal birds, it is not known whether the same song sung by day and at night is directed to the same receiver (male, female, or both) and if the dominant function of the song (mate attraction or rival deterrence) changes in the day–night context. Several hypotheses have been proposed to explain the function of nocturnal singing in diurnal birds [19]. The most convincing seem to be: (1) mate attraction (the male’s nocturnal singing increases the chances of attracting a female, including extra–pair copulations); (2) territory defence(nocturnal singing informs potential rivals about the occupation of the territory and readiness to defend it); (3) stimulation of reproduction (the male’s singing increases the production of hormones stimulating the production of a larger brood); (4) female defence (the male’s singing informs the female about his presence and induces the partner’s response, preventing extra–pair copulation by the female); (5) avoiding predators (nocturnal singing is limited in areas with high predator pressure at night, but nocturnal vocal activity is increased in areas with strong predator pressure during the day). The functions of nocturnal singing by diurnal birds are not mutually exclusive and may differ across taxa or ecological and environmental conditions [20, 21].

The function of nocturnal singing by daily active birds has been examined only in a few studies, focused on only a few species, yielding inconsistent results [10, 22]. Moreover, most of the studies considered only the advantages of night singing and ignored the potential costs of such behaviour. Singing at night by diurnally active birds seems to be risky and may decrease the survival rate–an individual singing at night is easier to be detected by predators than a silent one and might have a lower chance of detecting an approaching predator. Thus, night singing by diurnal birds should occur only when the benefits from increased reproductive success outweigh the costs of a lower survival rate for night singers. If nocturnal singing increases reproductive success, we should expect that the appearance of such behaviour in some individuals from the population would elicit reactions from their conspecifics. Otherwise, the absence of behavioural responses would suggest that night singing by diurnal species serves no function. To fully understand the evolution and mechanisms of night singing by diurnal birds, there is a need for a larger number of experimental studies conducted under various ecological conditions and focused on species that sing at night both occasionally and regularly.

In this study, we focused on night singing in two typical diurnal songbirds–the yellowhammer (*Emberiza citrinella*) and the common chaffinch (*Fringilla coelebs*). These two similarly sized species commonly breed in Europe in different environments: the yellowhammer inhabits farmland, while the common chaffinch is a typical forest species. The territory size varies dependent on habitat type, but on average does not exceed 1 ha in farmland [23, 24]. Moreover, both species are monogamous and territorial, so they strongly respond to intrusion into the territory imitated by broadcasting conspecific songs from a loudspeaker [25, 26]. Males of both species sing during the breeding season. In the yellowhammer, the peak of the singing activity occurs at sunrise and sunset [27], while in the common chaffinch, there are no diurnal peaks of singing [28]. Both species rarely sing single songs at night, and so can be used to test the potential benefits and costs of night singing. The small number of night songs in these species might result from a lack of specific function of this behavior, but on the other hand, nocturnal singing can also be limited by ecological factors.

The aim of the study was to determine whether night singing in species which normally do not sing at night elicits responses from their conspecifics. We conducted an automated field playback experiment, in which we broadcast songs of a foreign male to the territory holder during both day and night, at the beginning and in the middle of the breeding season. We predicted that if nocturnal singing had no function, birds would ignore night playback and respond only to the daytime intrusion. Otherwise, a response to the night playback would suggest that night singing may increase reproductive success but can be limited by ecological factors. Since males’ singing intensity and aggressiveness toward intruders vary both daily and seasonally, we expected a more aggressive response at the beginning (mating time) than in the middle of the breeding season. Moreover, we hypothesised that birds would respond more intensely to night playbacks before the female arrives on the territory during the premating stage than after the females arrive and the location of the female is well established. This would support that night song’s function is to establish and maintain the pair bond.

We reasoned that any seasonal differences in birds’ day and night response patterns would give clues to potential differences in the function of day and night singing.

## Methods

### Study site

The study was conducted in Bialowieża Glade (eastern Poland, central Europe; coordinates: 52.70089N, 23.86763E)–the largest settlement glade in the Polish part of the Bialowieża Primeval Forest [29]. The glade covers approximately 13.5 km2 and serves as suitable breeding habitat for many bird species. The main part of the study site was covered by extensively managed farmland, with a large proportion consisting of meadows and arable fields undergoing secondary forest succession within the protective zone of the Bialowieża National Park [30]. The tested territories of birds were located outside of the village, on the forest edge, or in farmland buffer strips (S1 Fig). Therefore, the birds were not affected by anthropogenic noise and light pollution. The study site enabled us to test the function of night singing by diurnal birds in their natural ecological conditions.

### Field data

In each territory, we conducted a playback experiment during the early stage (from 20th to 28th April 2021) and the middle stage (19th to 25th May 2021) of the breeding season. During both periods, the phase of the moon was similar, and the percentage of moon illumination (the surface of the moon visible in the sky) ranged from 50% to 99%. The moon was always visible at night and the sky was clear during the playbacks. We tested 14 yellowhammer territories and 17 common chaffinch territories. The experiments were conducted during the nautical night (the period when the sun is at least 12 degrees below the horizon; from 10:05 PM to 02:04 AM) and repeated on the same territory during the day (after sunrise; from 4:48 AM to 9:15 AM). Playback experiments were done in the rotated order: part of them in the order of night followed by day and part of them in the order of day followed by night. This approach eliminates the effect of taming the bird with playback. In our experiment, we did not recognise birds individually; therefore, different males may have occupied the territory at different stages of the breeding season. To avoid pseudo-replication and bird stimulation at the local level, territories within 300 meters of each other were tested on different days.

To minimise the effect of the observer on the bird’s behaviour and to make comparable measures of bird response during both day and night, the experiment was conducted completely automatically. Firstly, the experimental area in the place from which the territory holder sings the most often was determined. For this purpose, a few days before the experiment, maps of estimated territory boundaries through visual and acoustic observations of birds in the morning were drawn. The distance between the nearest neighbouring territories of the same species varies between 50m to 800m, but in the same day we were used territories separated exceed 300 m. We placed a loudspeaker (Ultimate Ears Boom 2) connected to the player (Olympus LS–P1) on a tree or shrub in the middle of the experimental area, approximately 2 m above the ground level. Three meters from the loudspeaker, one camera trap (Camera Trap Full HD 40 IR + GPRS MMS) was placed on a pole (1.5 m above the ground), which recorded birds’ behaviour during the experiment. Additionally, the vocal response of the bird in the tested territory was recorded using four automatic acoustic recorders (AudioMoth 1.1.0; 48 kHz/16–bit sampling rate) located on trees or shrubs. The recorders were placed on a triangular-like plan (arranged along imaginary lines that were 120° apart from each other), on trees or shrubs, at a height of 2–3m. One recorder was placed 2–5 m from the speaker, and the other three were placed up to 46 m from the speaker. In this way, it was possible to achieve a high signal-to-noise ratio for the vocal responses of birds located in different parts of the territory. Experiments started automatically, without the presence of researchers in the experimental area, and comprised three phases: (1) 20 minutes before the playback (silence); (2) 20 minutes of the playback broadcast from the speaker; (3) 20 minutes after the playback (silence).

### Playback design

**Fig 1:**
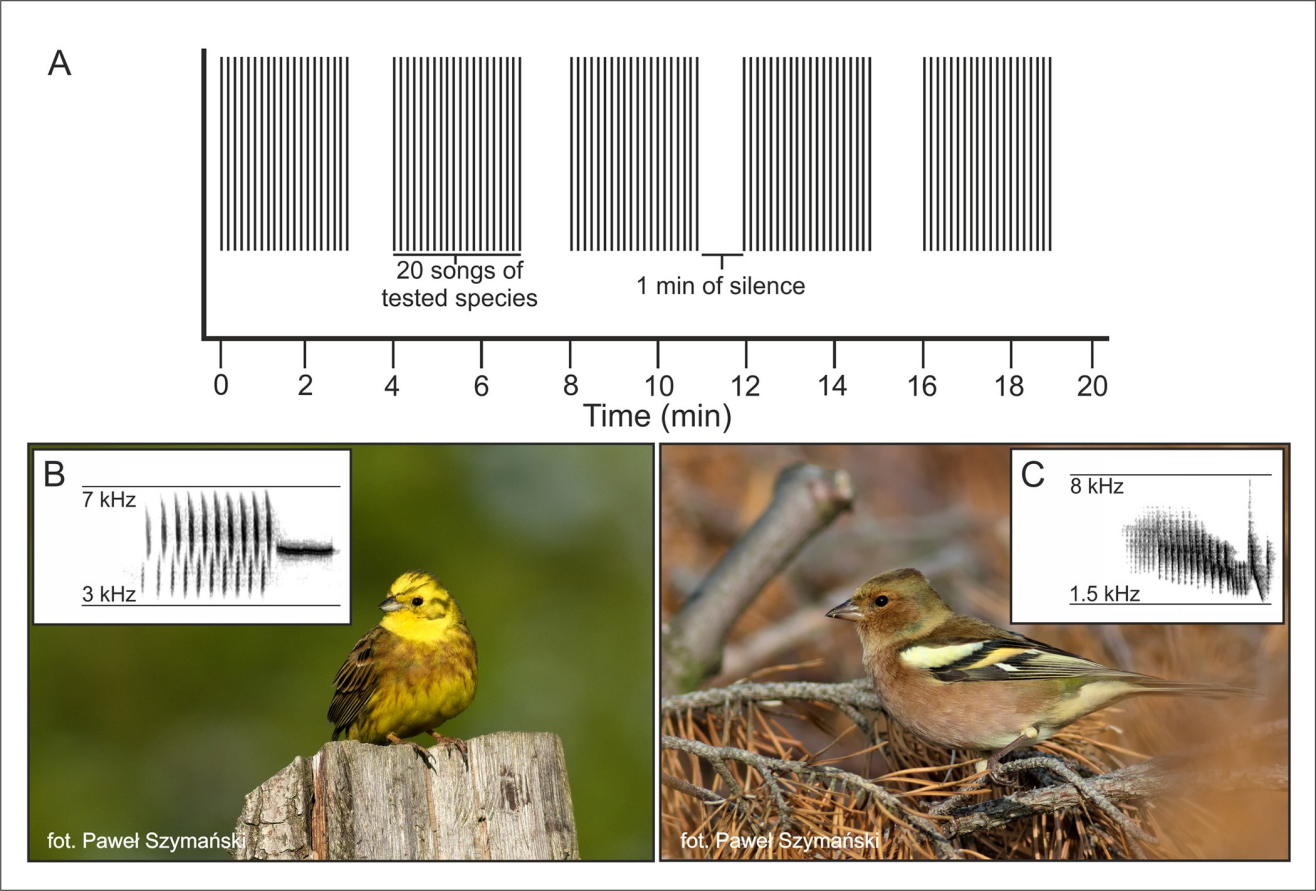
**A) Visualizations of playback design.** The treatment contained five series of 20 songs separated by 1–min silence. **B) Photo of the yellowhammer and a spectrogram of its typical song. C) Photo of the common chaffinch and a sonogram of its typical song**.

We prepared 10 sound samples of each species to use in the experiment. To prepare each sound sample, high–quality recordings of 10 different males collected in Greater Poland and Masovian Voivodship were used. This ensured that the males in the tested territories were not familiar with the playback song samples. Every playback lasted 20 minutes and consisted of five three–minute sections (normal singing rate for both species during the day) separated by one minute of silence (Fig 1). Each section contained 20 high–quality songs from the same male (10 different songs repeated two times). Audio files were prepared using Avisoft SASLab Pro 5.2.12 software by one person (KB). For each playback, a high–pass filter (700 Hz for the common chaffinch and 1 500 Hz for the yellowhammer) was applied to remove background noise. This cutoff does not overlap with the song frequency of either species. Additionally, the volume of the playback was normalized by setting the SPL level at 85 dB (+/– 2dB; measured at 1 m by using UNI–T UT351 sound level meter), which is the average volume of the singing birds [31].

### Acoustic and video analyses

The vocal responses recorded by automatic acoustic recorders were analysed using Avisoft SASLab Pro 5.2.12 software. To generate spectrograms, we applied a 1024 FFT Length, 75% Frame, and Hamming Window. Each song recorded during the experiment was classified to the species, and the time of the beginning of each song (in seconds) was noted during manual scanning of spectrograms and listening to the recordings.

Video recordings showing the flight response of individuals in the tested territories were collected by infrared camera traps capable of making recordings at night. Each of the 60 one–minute videos from the entire experiment was analysed using DaVinci Resolved software. Every movie was accelerated 10 times, and every movement of birds or predators was noticed and classified to species.

Finally, we used two measures of bird response: the number of songs produced during each phase of the experiment and the number of flights recorded during each part of the experiment. Due to the insufficient quality of the videos and the quick movement of birds, it was not possible to recognize the sex of the individuals on the video recordings either during the day nor the night.

We collected the full audio data set (day and night experiments with 3 phases conducted twice in a season) for all 14 territories of the yellowhammer and all 17 territories of the common chaffinch

Due to a technical problems (the batteries in camera traps discharged very quickly) we collected an incomplete video data set, which is presented in Table 1.

**Table 1:** Volume of complete video data (before playback phase, playback phase, after playback phase) within different stages of the breeding season (April, May) and time of experiment (day, night). N = number of all tested territories.

	Yellowhammer (n = 14)		Common chaffinch (n = 17)	
	*April*	*May*	*April*	*May*
**Day**	12	8	11	14
**Night**	10	11	7	13

### Statistical analyses

Since no vocal response occurred during the night part of the experiment for both species, we analysed only the control, daily part of the experiment, to check whether the playback stimulates birds to produce vocalisations. We compared the numbers of songs and calls produced by both species before, during, and after the playback using Generalized Linear Mixed Models (GLMMs) with negative binomial family. This approach let us estimate the parameters for count data, while controlling for overdispersion. We built two separate models for each species with the number of songs or calls as a response variable, experiment phase (before, during, and after playback as the three–level category) and stage of the breeding season with interaction between them as fixed effects, and territory ID of the experiment as random intercept effect, day/night was not included as a variable as no vocalizations were recorded at night. The interaction between fixed effects was kept or dropped based on Akaike Information Criterion (AIC) tested with Chi-square test. We lack information whether birds in territories were the same individuals during the experiment in both stages of the breeding season. A possible change of territory may occur when the nest is destroyed and the pair will have to rebuild it in another place, however, focal birds are likely the same individuals. On the other hand, each territory is unique in terms of the predatory pressure, biocenosis and abiotic environmental parameters which influence sound propagation. Therefore, we treated each territory in our analysis separately which was implemented as a random intercept effect.

To assess whether the birds showed flight responses to the vocal stimulus of foreign males, we compared the probability of flights before, during, and after the playback, separately for each species. These models contained the probability of flight as a response variable, the stage of breeding season (two–level factor–April and May), experiment phase (before, during, and after playback as the 3–level category), time of the experiment (two–level factor–day and night) with the interaction between the experiment phase and the time of the experiment as fixed effects, with an ID of territory nested in month as a random intercept effect. We avoided testing three–way interactions (experiment phase with a time of experiment to month) using one conditional model due to over-parameterization. The count data of flights were converted to binary since most observations with >1 flight were observed only for playback during the day category (57% for the common chaffinch and 66% for the yellowhammer), while the variance of other groups was low. The most important information was whether or not the birds responded at night, rather than the intensity of the responses.

All models were implemented in R using the glmmTMB package [32] and built–in functions. For negative binomial models the log link function was used, while for the binomial one it was the logit link function. The overall effects of the fixed variables on the response were assessed using Type–II or Type–III Wald chi–square tests (using Car package; [33],) and the choice of II or III SS was selected based on the significance of the interaction [34]. The results were visualised using partial residuals with visreg and ggplot2 packages [35, 36]. Data and the R code are available under the link: https://github.com/kinkul1/The-response-of-two-European-species-of-birds-to-singing-of-foreign-male-at-night.git.

### Ethics approval

Methods in this research were approved by the Regional Directors for Environmental Protection in Bialystok, Poland (number of permission: WPN.6401.41.2021.DO). The implementation of the experiment did not pose a threat to the wild population of the yellowhammer or the common chaffinch.

## Results

### Vocal response

No vocal responses during the nocturnal playback experiments by either yellowhammers or common chaffinches were observed. 246 songs and 180 calls of the common chaffinch and 296 songs and 250 calls of the yellowhammer were recorded during the day across all phases (before the playback, during the playback, and after the playback) in two recording seasons (April and May). More details are presented in Table 2.

**Table 2:** Comparison of the average number of songs and calls between the experimental phases during the day and night. The values in brackets are standard errors of the means.

		Common chaffinch				Yellowhammer			
		Day		Night		Day		Night	
		Songs	Calls	Songs	Calls	Songs	Calls	Songs	Calls
*April*	Before	24 (8.8)	10 (7.6)	0	0	44 (7.5)	11 (7.3)	0	0
	Playback	30 (9.2)	41 (15.5)	0	0	45 (12.7)	145 (36.1)	0	0
	After	49 (49.9)	62 (17.6)	0	0	75 (9.8)	14 (6.5)	0	0
*May*	Before	36 (13.0)	9 (4.5)	0	0	33 (10.7)	0	0	0
	Playback	47 (13.1)	40 (13.5)	0	0	39 (18.5)	75 (30.3)	0	0
	After	60 (11.4)	18 (6.2)	0	0	60 (14.9)	5 (4.1)	0	0

**Fig 2:**
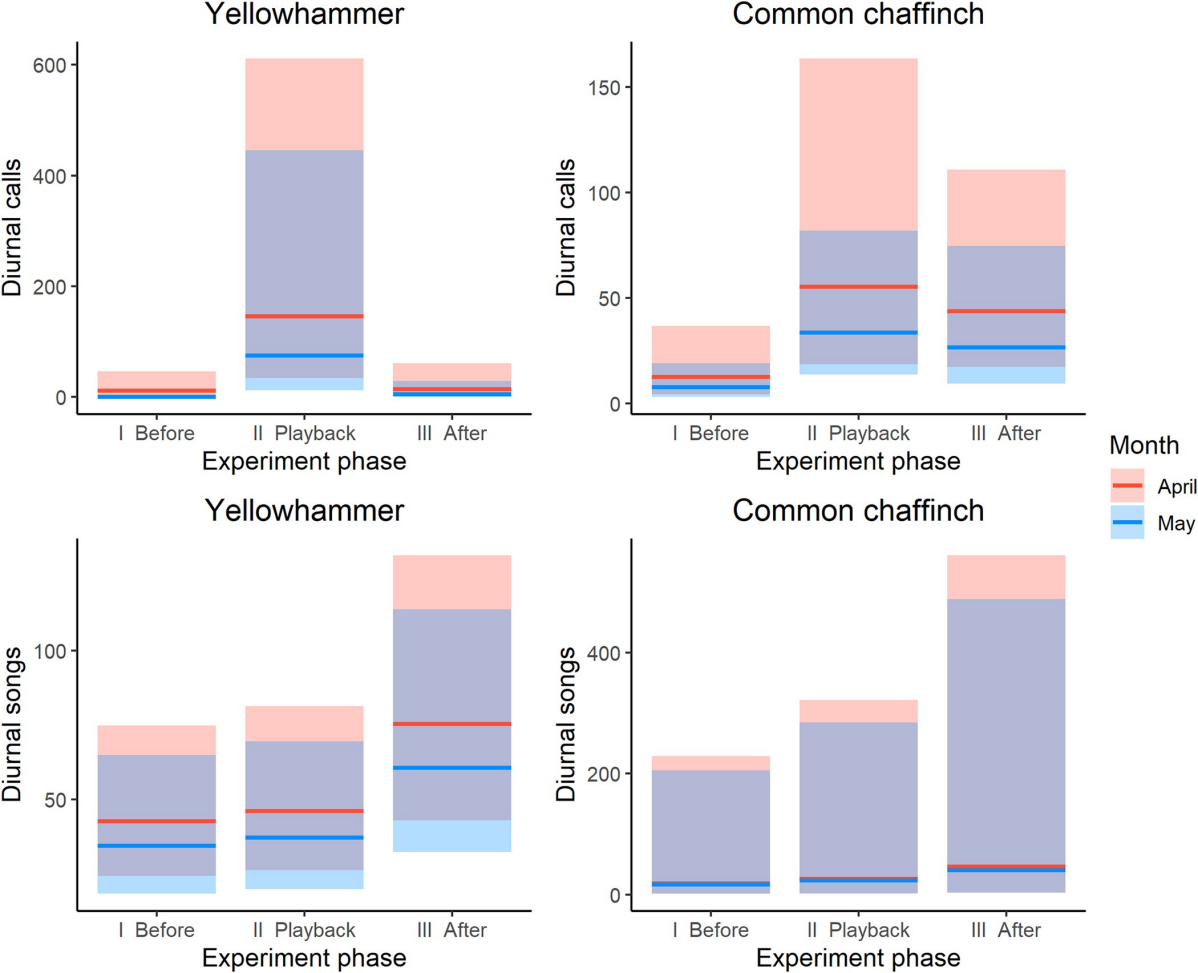
The number of diurnal calls and songs produced by the yellowhammer and the common chaffinch at different stages of the breeding season and in different experiment phases (before, playback, after). The graph represents only daytime results because birds did not respond vocally during night-time experiments for both species. Lines–the estimate; bars– 95% confidence intervals. (2–column fitting image). *Confidence intervals for calls of the yellowhammer during May before playback is undefined (only 0s observations).

During the daytime playback experiments, the song rate of the yellowhammer did not depend on the playback phase (χ2 = 2.791, df = 2, p = 0.248) and was not dependent on the stage of the season (χ2 = 0.489, df = 1, p = 0.484); the model with interaction has no significantly better goodness of fit than the additive model (χ2 = 0.011, df = 2, p = 0.995). However, the number of calls yellowhammers produced during the experiment depended on the experiment phase (χ2 = 11.917, df = 2, p = 0.003)–birds produced significantly more calls during the playback than before or after it (S1 Table, Fig 2). Nevertheless, even if the model with the interaction of the stage of the breading season with experiment phase showed significantly better goodness of fit (χ2 = 6.867, df = 2, p = 0.032), the overall effects of the breeding stage of calls were minor (χ2 = 1.145, df = 1, p = 0.285).

The number of songs produced by common chaffinch males in response to the playback was independent of the phase of the experiment (χ2 = 5.755, df = 2, p = 0.056; S 2) and the stage of the breeding season (χ2 = 0.046, df = 1, p = 0.831; S 2). Moreover, the model with interaction did not show significantly better goodness of fit than the additive model (χ2 = 0.162, df = 2, p = 0.922). We did not find a significant effect of the stage of the season on the number of calls produced by chaffinches (χ2 = 0.947, df = 1, p = 0.331) independently of whether the interaction between fixed effects was included or not (χ2 = 1.115, df = 2, p = 0.573). However, the number of calls produced by chaffinches varied between experiment phases (χ2 = 6.856, df = 2, p = 0.032). Chaffinches produced significantly more calls during and after the playback in comparison to before the playback phase. See Fig 2 and S1 Table for data and model results.

### Flight response

**Fig 3:**
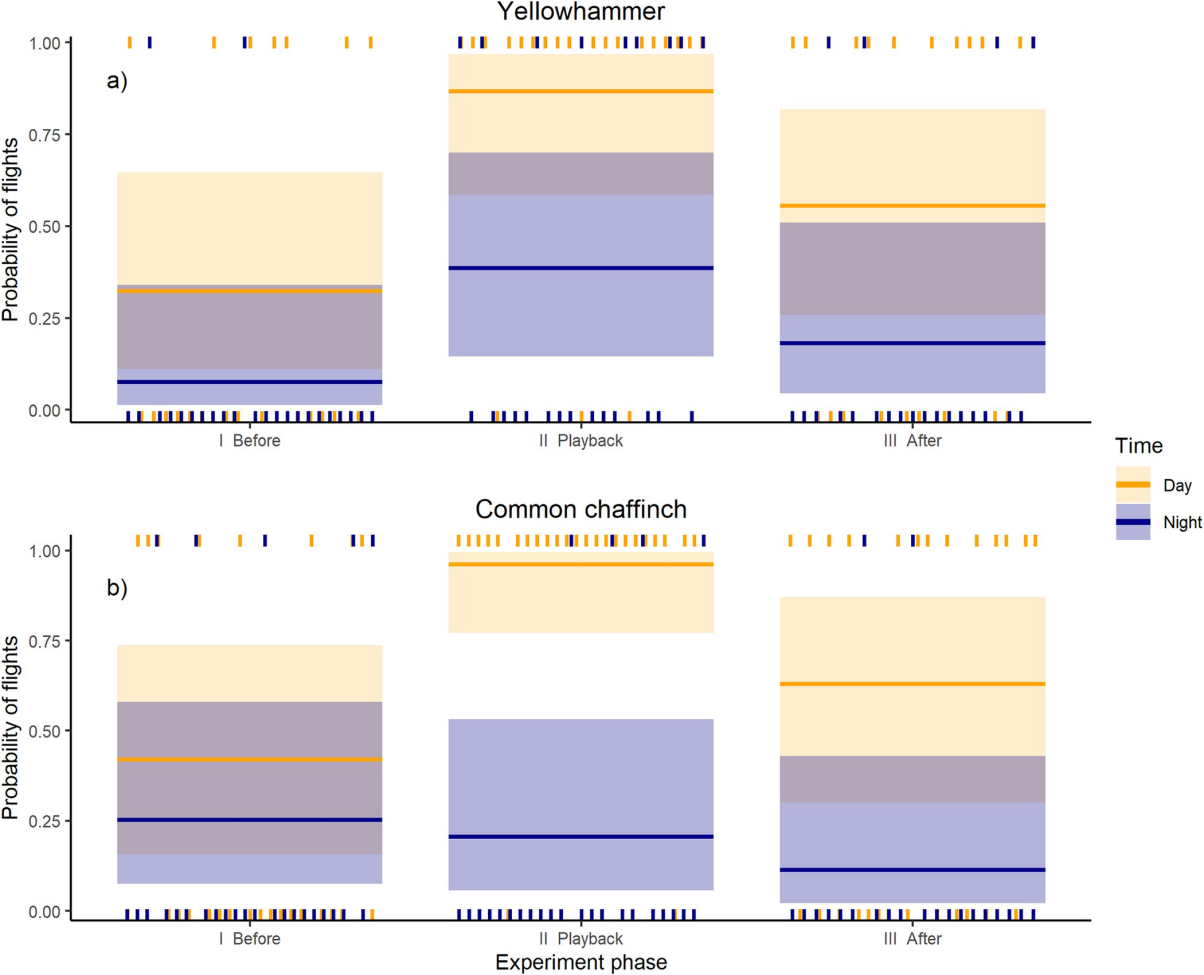
Probability of flights during the day (red plot) and night (blue plot) in different experiment phases (before, playback, after) in the yellowhammer and the common chaffinch. Shadowed colour blocks represent 95% confidence intervals, while dashes represent observations that were higher (at the top) or lower (at the bottom) than 50% probability threshold. Colours represent time in the day, red represents daytime, while blue represents night. (1,5–column fitting image).

We observed yellowhammers flying near the speaker in each phase of the experiment, both during the day and at night. The probability of flying was independent of the stage of the breeding season (χ2 = 0.196, df = 1, p = 0.658). Yellowhammers were more likely to be observed flying near the speaker during the day than at night (χ2 = 15.427, df = 1, p < 0.001). Moreover, the probability of flying was related to the experiment phase (χ2 = 14.913, df = 2, p < 0.001) and was more probable during and after the playback than before it (Fig 3 and S1 Table). However, we did not find a significant interaction between the phase of the experiment and the time of the day (χ2 = 0.393, df = 2, p = 0.822), which means that the general pattern of bird behaviour before, during and after the playback is similar during the day and night–the yellowhammer is more likely to take an action as flight during and after the playback than before it.

During the day, the probability of the common chaffinch’s flights through and after the playback was higher than before (S2 Table and Fig 3). The significant effects of the time of the day (day vs night; χ2 = 19.493, df = 1, p < 0.001), with a significant interaction between the time of the day and the phase of the experiment (χ2 = 10.126, df = 2, p = 0.006) means that birds responded to the playback only during the day, not during the night. The overall probability of flight was similar for both stages of the breeding season (χ2 = 2.713, df = 1, p = 0.100). The details and visualisations of the models are presented in S 3 and Fig 3. Overall, the common chaffinch responded to the playback only during the day, while during the night we did not observe any significant changes in the probability of flights between different stages of the experiment.

### Predator pressure

During the nocturnal part of the experiment, predators moving directly to the loudspeaker were recorded. In the case of the yellowhammer, the tawny owl (*Strix aluco*) was observed twice at the same territory during the same night (during playback and after playback). It was 7% of territories in which predators were observed during playback. For the common chaffinch, the tawny owl was observed six times in three different territories, and pine martens (*Martes martes*) were recorded four times in three different territories (Table 3). It was 35% of territories in which predators were observed during playback. In one territory of the yellowhammer and three territories of the common chaffinch predators were seen during the playback phase and again after the playback. Among them, there was one attempted attack, with the tawny owl attacking the loudspeaker during the playback. Predators were not observed during the experiment conducted by day.

**Table 3:** The number of the appearances of predators during the nocturnal experiment.

Experiment phase / Species	Yellowhammer	Common chaffinch
**Before playback**	–	1 Pine marten
**During playback**	1 Tawny owl	2 Pine martens 3 Tawny owls
**After playback**	1 Tawny owl	1 Pine marten 3 Tawny owls

## Discussion

Our results showed that playback of songs from foreign males did not stimulate either of the tested species to sing or produce calls at night, in contrast to the diurnal playback experiment. During the day, a response typical of many songbirds towards simulated acoustic intrusion was observed [37, 38]. Both species produced more alarm calls during the playback stage than before the playback, while the number of songs did not differ between the playback stages. This pattern may result from a completely automatic experimental approach–the playback was turned on independently of the birds’ vocal activity. In a typical experimental design, the playback is usually turned on when the tested male is singing. Thus, the highest number of songs is observed before the playback, while during the playback stage birds switch from singing to calling and flying near the speaker [26, 39].

In our study, we did not observe yellohammers and chaffinches singing at night, neither before, during, nor after the playback of conspecific songs. It is possible that birds ignore the playback of foreign males because nocturnal singing has no function for them. The lack of vocal response at night may be also due to the birds’ not feeling threatened by the singing of conspecific males, since the possibility of intruders singing at night is low.

Another proposed explanation for the absence of nocturnal singing is that high predator pressure can limit this behaviour. We observed that the nocturnal playback of the yellowhammer and the common chaffinch attracted predators like tawny owls and pine martens multiple times, which was not observed during the day. Predators were seen in widely scattered territories, therefore predation pressure is likely to be important for all individuals (S1 Fig).

These predators rely mainly on hearing to track their prey, which is often small-sized passerines [40]. Therefore, a single individual singing in the silence at night seems to be an easy target. If higher frequency of predator observations during chaffinch’s trials (35% of territories in which predators were observed during playback) reflects greater predator pressure, then in the common chaffinch’s breeding areas this may have contributed to lower flight response during the playback, while the less frequently observed predators in the yellowhammer’s territories (7% of territories in which predators were observed during playback) may be responsible for the higher flight response during nocturnal playback by this species. Multiple studies have shown that high predator pressure is a factor limiting singing in diurnal birds. Veery (*Catharus fuscecens*) males sang less intensely after dusk when owl vocalizations were played back to them, mimicking the heavy predator pressure at night [41]. Santema et al. found that many species that normally sang at dawn were less likely to do so after hearing sounds of predators, and when they did sing they started later than typically [42] Our study suggests that ecological factors, such as predator pressure may be possible explanation why birds limit their singing at night.

In contrast to the vocal response, in both tested species, movements during both diurnal and nocturnal playback were observed. However, the pattern of response differed between the species. Yellowhammers flew more during the diurnal playback than the nocturnal one, but the pattern of response was similar during both parts of the day. For both periods (day and night), the playback of foreign males stimulated yellowhammers to a higher flight response than before the vocal stimuli. The flights at night consisted of sporadic single, very quick displacements in the vicinity of the loudspeaker, while during the day they involved very fast movements near the loudspeaker, flying from branch to branch. Both responses were aggressive and could imitate an attack to deter intruders from the territory. This suggests that in yellowhammers, both diurnal and nocturnal songs pose a similar threat to the territory holder; however, the intensity of the nocturnal response may be limited by the ecological costs of such risky behaviour under low visibility conditions. The constant flight response across the season (April and May) also indicates that birds strongly defend the territory during this time.

In common chaffinches, the pattern of flight response differed between day and night. During the day, birds flew more than at night and, similar to yellowhammers, showed the highest probability of flight during the playback stage. At night, we observed no differences in the probability of flights between the experimental phases. The lack of flight response in the common chaffinch at night may be related to the high predation pressure in its territories. Alternatively, the ability to see under low levels of light may be lower in this species, which should weaken the response, or the motivation to defend resources, such as territory or females, may be significantly lower at night.

Other experimental studies demonstrated different functions of nocturnal singing. For example, in the ovenbirds (*Seiurus aurocapilla*), males do not counter-sing to the playback of foreign males at night, and the explanation for this behaviour is that nocturnal singing is directed towards females [21]. Similarly, male field sparrows (*Spizella pusilla*) do not respond by singing to the vocal stimulation from an unknown male but move more often, with the intensity of the reaction remaining constant during the breeding season [17]. In contrast, female mobility in response to a foreign male’s singing was highest before and during its most fertile period, which may support the mate attraction hypothesis [20]. In the common nightingale (*Luscinia megarhynchos*), night singing is primarily performed by unpaired individuals. This activity ceases after finding a partner but is reactivated when the female leaves the male. This suggests that unpaired male nightingales use intense night singing to attract mates [7]. On the other hand, the constant vocal activity of the common nightingale during the dawn chorus, independent of the reproductive period of the female, indicates that during the day, the proposed function of singing is to defend the territory [43]. The discrepancy in the proposed functions of nocturnal singing observed in different studies of diurnal bird species may indicate that the possible function is species- and environment-specific. Our study suggests that predator pressure may limit night singing. Future work should consider environmental variables affecting risks associated with singing in other species and circumstances.

## Supporting information

S1 Fig The map showing the distribution of the tested territories and spatial distribution of predator pressure. (TIF)

S1 Table Results of GLMMs testing differences in vocal responses of the yellowhammer and the common chaffinch at different stages of the breeding season (early and middle) and different experimental phases (before, playback, after). (DOCX)

S2 Table Results of GLMMs testing differences in the probability of flights performed by yellowhammers and common chaffinches during the day and night in different parts of the breeding season and different experiment phases (before, playback, after). (DOCX)
