# Supplementary figures and images for: Nocturnal playback experiments: The response of two European species of birds to singing of foreign male at night
Source: PLoS One. 2024 Nov 25;19(11):e0313427. doi: 10.1371/journal.pone.0313427 (PMC11588260; doi:10.1371/journal.pone.0313427)

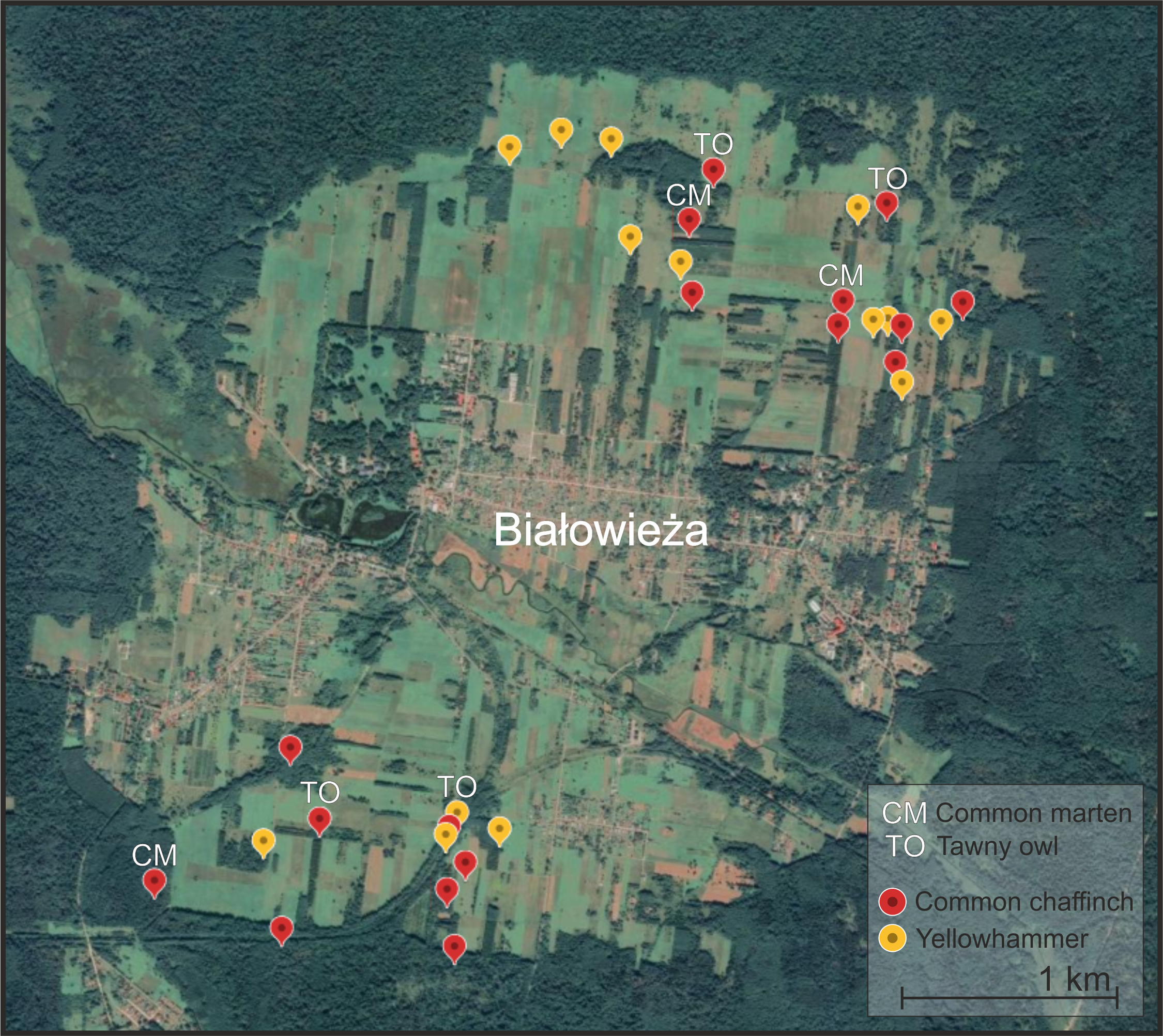

Supplement: S1 Fig — (TIF) [file pone.0313427.s001.tif]
