# Supplementary material for: Nocturnal playback experiments: The response of two European species of birds to singing of foreign male at night
Source: PLoS One. 2024 Nov 25;19(11):e0313427. doi: 10.1371/journal.pone.0313427 (PMC11588260; doi:10.1371/journal.pone.0313427)
Supplement: S1 Table — (DOCX) [file pone.0313427.s002.docx]

**S 2** Results of GLMMs testing differences in vocal responses of the yellowhammer and the common chaffinch at different stages of the breeding season (early and middle) and different experimental phases (before, playback, after).

|  | **Estimate** | **SE** | **z** | **p** |
| --- | --- | --- | --- | --- |
| ***Yellowhammer – songs*** | | | | |
| Intercept | 3.831 | 0.290 | 13.232 | <0.001 *** |
| Phase [Before] | –0.078 | 0.367 | –0.210 | 0.834 |
| Phase [After] | 0.490 | 0.369 | 1.329 | 0.184 |
| Month [May] | –0.216 | 0.309 | –0.669 | 0.484 |
| ***Yellowhammer – calls*** | | | | |
| Intercept | 4.983 | 0.731 | 6.820 | <0.001 *** |
| Phase [Before] | –2.579 | 1.036 | –2.489 | 0.0128 * |
| Phase [After] | –2.309 | 1.036 | –2.230 | 0.0258 * |
| Month [May] | –0.670 | 1.168 | –0.573 | 0.566 |
| Phase [Before]: Month [May] | –27.96 | inf | 0.000 | 1.000 |
| Phase [After]: Month [May] | –0.440 | 1.660 | –0.265 | 0.791 |
| ***Common chaffinch – songs*** | | | | |
| Intercept | 3.271 | 0.483 | 6.773 | <0.001 *** |
| Phase [Before] | –0.338 | 0.376 | –0.901 | 0.367 |
| Phase [After] | 0.557 | 0.366 | 1.522 | 0.128 |
| Month [May] | –0.127 | 0.569 | –0.213 | 0.831 |
| ***Common chaffinch – calls*** | | | | |
| Intercept | 4.014 | 0.553 | 7.261 | <0.001 *** |
| Phase [Before] | –1.479 | 0.606 | –2.442 | 0.0146 * |
| Phase [After] | –0.233 | 0.620 | –0.375 | 0.707 |
| Month [May] | –0.502 | 0.516 | –0.973 | 0.331 |
