# Supplementary material for: Nocturnal playback experiments: The response of two European species of birds to singing of foreign male at night
Source: PLoS One. 2024 Nov 25;19(11):e0313427. doi: 10.1371/journal.pone.0313427 (PMC11588260; doi:10.1371/journal.pone.0313427)
Supplement: S2 Table — (DOCX) [file pone.0313427.s003.docx]

**S3** Results of GLMMs testing differences in the probability of flights performed by yellowhammers and common chaffinches during the day and night in different parts of the breeding season and different experiment phases (before, playback, after).

|  | **Estimate** | **SE** | **z** | **p** |
| --- | --- | --- | --- | --- |
| ***Yellowhammer*** | | | | |
| Intercept | –0.374 | 0.501 | –0.736 | 0.462 |
| Phase [Before] | –2.033 | 0.875 | –2.323 | 0.020 * |
| Phase [After] | –1.040 | 0.722 | –1.441 | 0.150 |
| Time [Day] | 2.234 | 0.805 | 2.905 | 0.004 ** |
| Month [May] | –0.213 | 0.481 | –0.443 | 0.658 |
| Phase [Before]: Time [Day] | –0.581 | 1.177 | –0.494 | 0.621 |
| Phase [After]: Time [Day] | –0.612 | 1.063 | –0.575 | 0.565 |
| ***Common chaffinch*** | | | | |
| Intercept | –2.323 | 0.708 | –3.281 | 0.001 ** |
| Phase [Before] | 0.259 | 0.765 | 0.338 | 0.735 |
| Phase [After] | –0.703 | 0.947 | –0.743 | 0.458 |
| Time [Day] | 4.563 | 1.034 | 4.415 | <0.001 *** |
| Month [May] | 0.840 | 0.510 | 1.647 | 0.010 |
| Phase [Before]: Time [Day] | –3.796 | 1.204 | –3.153 | 0.002** |
| Phase [After]: Time [Day] | –1.980 | 1.284 | –1.542 | 0.123 |
